# Supplementary material for: Enhancing the thermostability and activity of glycosyltransferase UGT76G1 via computational design
Source: Commun Chem. 2023 Dec 6;6:265. doi: 10.1038/s42004-023-01070-6 (PMC10700650; doi:10.1038/s42004-023-01070-6)
Supplement: Supplementary file 2 — Supplementary Information [file 42004_2023_1070_MOESM2_ESM.pdf]

# Supplementary Information

# Enhancing the Thermostability and Activity of Glycosyltransferase UGT76G1 via Computational Design

Seong-Ryeong Go[1,2*], Su-Jin Lee[1,2*], Woo-Chan Ahn[1], Kwang-Hyun Park[1,2**] and Eui-Jeon Woo[2,3**]

[1]Critical Diseases Diagnostics Convergence Research Center, Korea Research Institute of Bioscience and Biotechnology (KRIBB), Daejeon 34141, Republic of Korea

[2]Department of Proteome Structural biology, KRIBB School of Bioscience, University of Science and Technology (UST), Daejeon 34113, Republic of Korea

[3]Disease Target Structure Research Center, Korea Research Institute of Bioscience & Biotechnology (KRIBB), Daejeon 34141, Republic of Korea

* These authors contributed equally to this work

** Corresponding Author: Eui-Jeon Woo (ejwoo@kribb.re.kr) or Kwang-Hyun Park (ruuua@kribb.re.kr)

## Supplementary Table 1. Mutation profile of UGT variants

| Energy (kcal/mol) | Name | mutation rate | Mutation |
|---|---|---|---|
| -1384 | WT | 0% (0 residues) | |
| -1428 | 76_1 | 2.2% (10 residues) | Δ1~11/N34W/D162E/S180F/G205A/S241F/S253T/S274Y/D291E/V300K/G348Y/ |
| -1464 | 76_2 | 3.8% (17 residues) | Δ1~11/I29M/N34W/K52A/L101F/R140P/D162E/S180F/G205A/S241F/S253T/S274N/D291E/I295M/R297W/V300K/S305P/G348Y/ |
| -1463 | 76_3 | 4.0% (18 residues) | Δ1~11/I29M/N34W/K52A/R103D/S119A/R140P/D162E/S180F/G205A/S241F/S253T/S274N/D291E/I295M/R297W/V300K/S305P/G348Y/ |
| -1475 | 76_4 | 5.4% (24 residues) | Δ1~11/I29M/N34W/K52A/L101F/R103D/S119A/R140P/L149A/D162E/S180F/S192C/G205A/S241F/S253T/Q270K/S274N/D291E/I295M/R297W/V300K/S305P/G348Y/V366I/Q425E/ |
| -1475 | 76_5 | 13.9% (62 residues) | Δ1~11/I16V/I29M/N34Q/V35I/N49Q/K52A/K54D/E95K/H96N/A98E/R103D/E104C/S119A/S136E/R140P/L149A/H153R/A154C/V156T/S157A/D162E/E163D/S192C/Q198K/I199Q/G205A/K206R/A213N/S221T/K223E/T232Q/S241H/H249Y/T263S/Q266S/Q270K/S274N/E286A/D291E/I295M/R297W/V300A/D301N/K303G/S305P/G348P/I350V/V366I/L392W/G405E/N409K/D417E/E418K/Y422E/Q425E/V429R/Q432E/V436L/M439K/K440P/S450K/S453D/ |
| -1467 | 76_6 | 3.1% (14 residues) | Δ1~11/I29M/V35I/R140P/N151S/G181E/S192K/G205D/L246F/S274N/I295M/L329P/G348E/L379G/L392W/ |
| -1488 | 76_7 | 4.0% (18 residues) | Δ1~11/I29M/V35I/A111N/S119A/R140P/N151S/G181E/S192K/G205D/L246F/Q266S/S274N/R297W/L329P/G348E/L379G/L392W/V429C/ |
| -1477 | 76_8 | 5.4% (24 residues) | Δ1~11/I29M/V35I/K54N/G87F/P91Q/A111N/S119A/R140P/N151S/G181E/S192K/I203L/G205D/L246F/Q266S/S274N/D291E/I295M/R297W/L329P/G348E/L379G/L392W/V429C/ |
| -1475 | 76_9 | 6.0% (27 residues) | Δ1~11/I29M/N34W/V35I/K54N/P91Q/A111N/S119A/R140P/N151S/D162E/G181E/S192K/I203L/G205A/L246F/S253T/Q266S/S274N/D291E/I295M/R297W/V300K/L329P/G348E/L379G/L392W/V429C/ |
| -1472 | 76_10 | 16.8% (75 residues) | Δ1~11/I16V/I29M/N34R/V35I/Y37H/F46L/K52S/K54N/T55P/P91M/I93L/E95K/H96N/A98E/E100P/L101F/R103D/E104C/A111N/S119A/A130T/S132A/S136E/R140P/L149A/N151S/H153L/S157A/Q160L/D162Q/L164K/Q178P/G181E/S192K/Q198E/I203L/G205D/A213R/K223E/E224C/T232W/S241C/L246F/T251P/H260Q/T263S/Q266S/Q270K/S274N/T284M/S285A/D291E/I295M/R297W/V300A/D301N/K303N/S305P/G348P/I350V/V366I/L379G/L392W/G405E/A408E/N409R/D417E/Y422E/N426R/V429E/Q432E/V436E/S437C/M439K/S456L/ |

**Supplementary Figure 1: Conversion activity of WT and 9 variants using HPLC analysis**

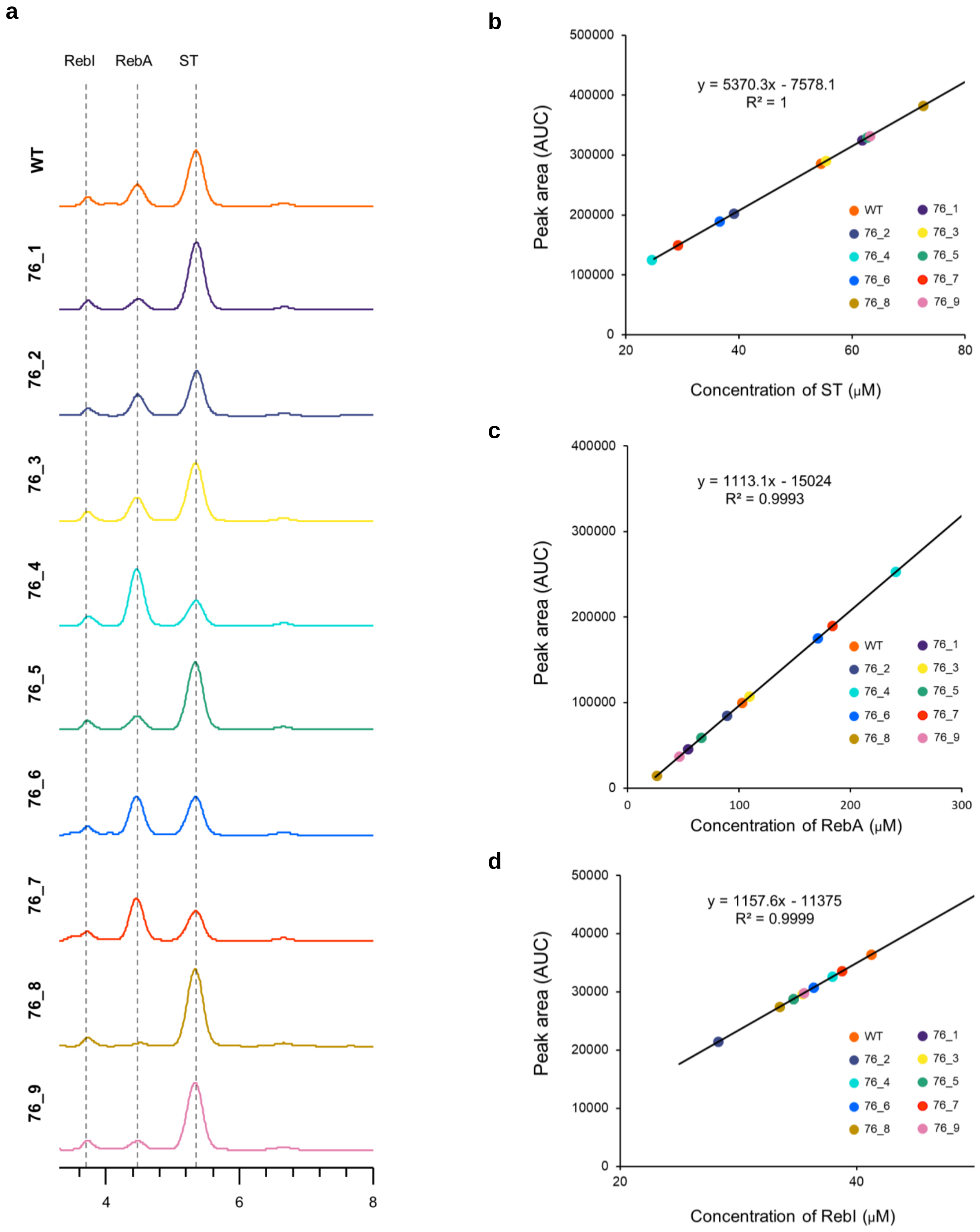


**a**, The complete set of chromatograms representing the activity screening of UGT variants toward ST, RebA and RebI analyzed using HPLC. The chromatograms include the wild-type (WT, represented in orange), and the variants (76_1 in purple, 76_2 in dark blue, 76_3 in yellow, 76_4 in cyan, 76_5 in bluish green, 76_6 in blue, 76_7 in red, 76_8 in brown, 76_9 in pink). **b-d**, Linear standard curve of ST, RebA, RebI and indication of substrate and reactant incubated with ST and UDPG in WT or the designed variants. The x-axis represents concentration of ST, RebA and RebI (uM) and the y-axis represents peak area at a 210 nm wavelength.

**Supplementary Figure 2: RebA conversion activity of variant 76_5 at higher temperatures**

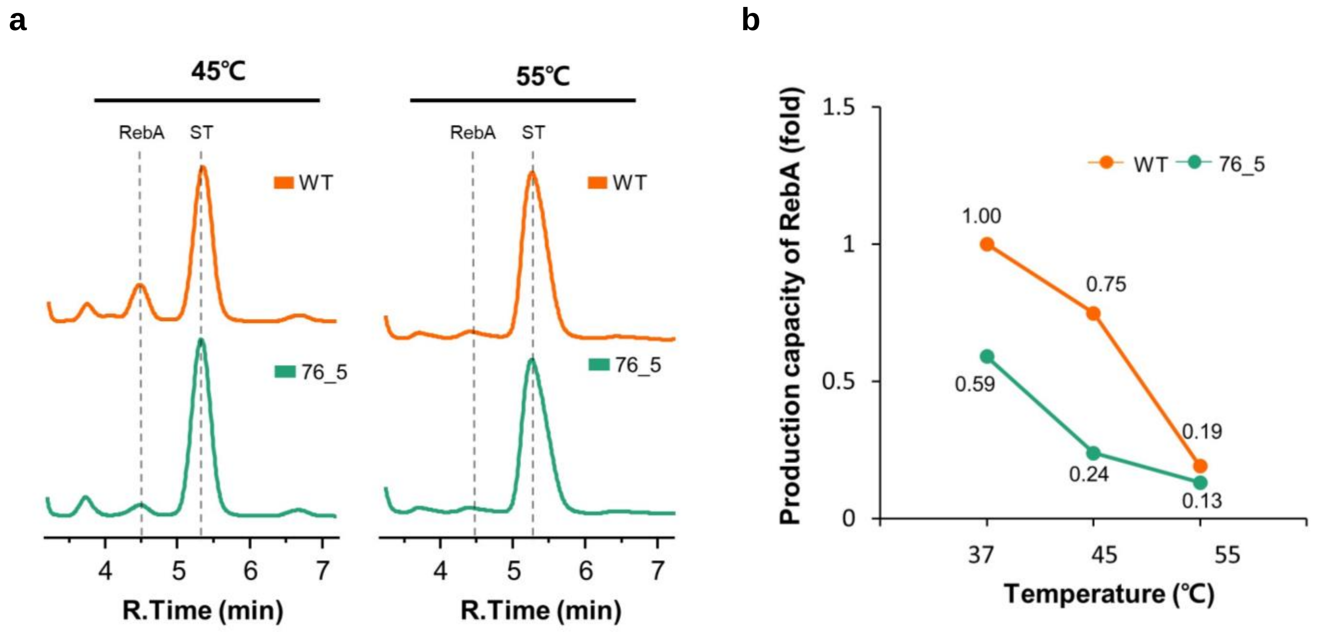


**a**, The chromatogram stack displays the profiles of the WT (orange), and variant 76_5 (bluish green). **b**, Production capacity of RebA of WT and 76_5 depends on temperatures.

**Supplementary Figure 3: Conversion activity of RebA to RebI by WT and variants**

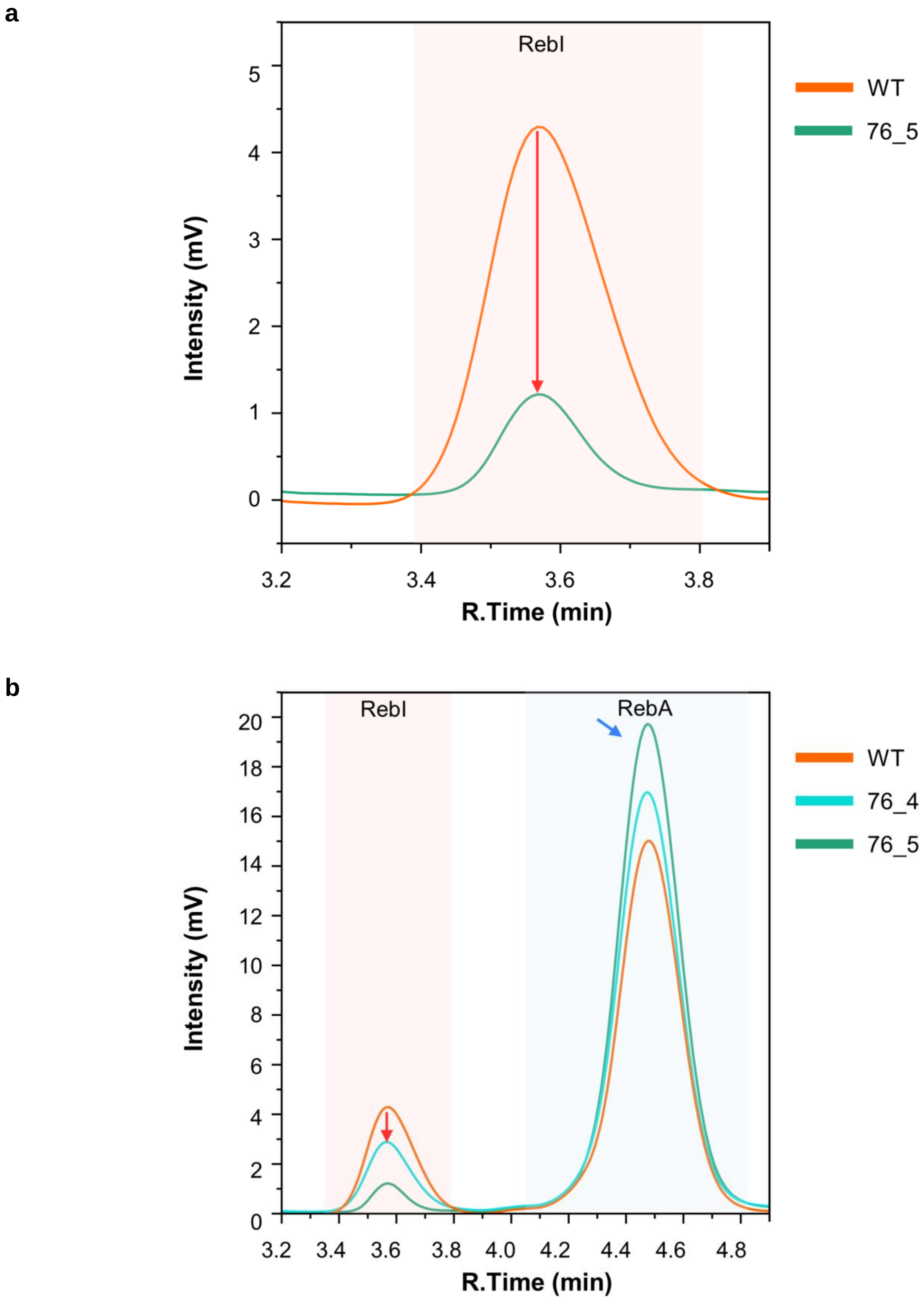


**a**, The chromatogram stack shows the RebI peak of the WT (orange), and variant 76_5 (bluish green). **b**, HPLC chromatogram of the conversion reaction of RebA to RebI catalyzed by WT, 76_4, and 76_5. The RebI peaks of 76_4 (cyan) and 76_5 (bluish green) were lower compared to that of WT (orange), while the RebA peak was increased in 76_4 and 76_5 compared to WT.

## Supplementary Figure 4: Stevioside docking simulation by SeeSAR

a b

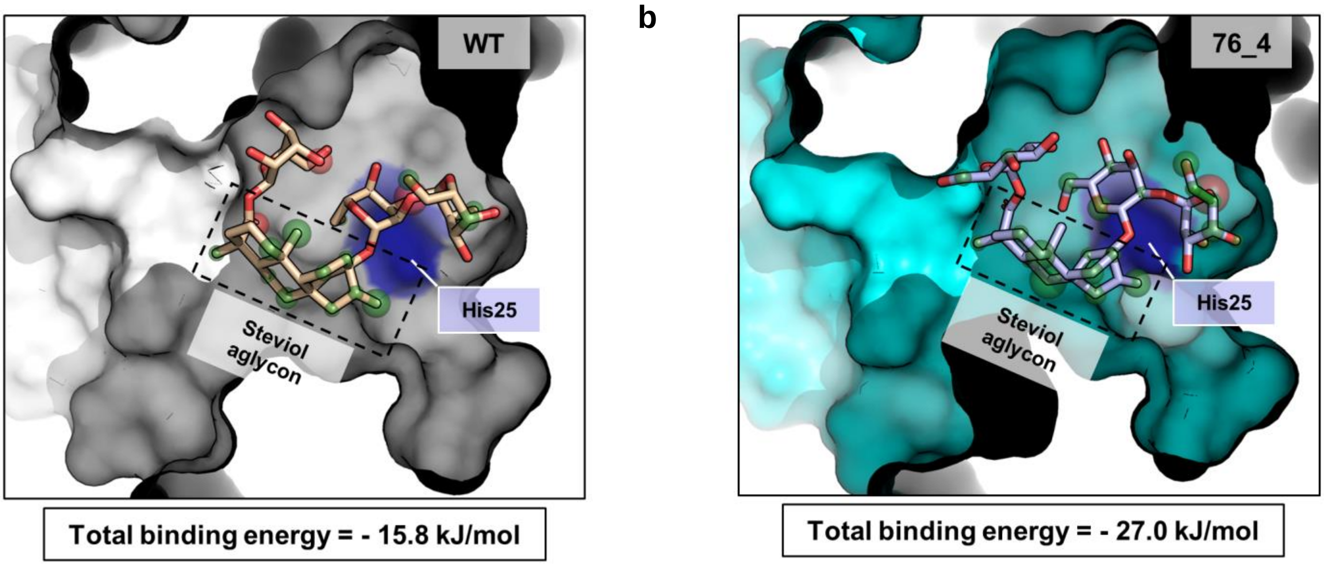


c

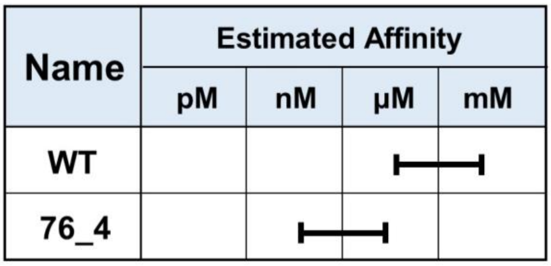


**a-b**, SeeSAR docking models of WT (white) with stevioside (wheat, ST) and variant 76_4 (cyan) with ST (light blue), respectively. The steviol aglycons are highlighted with black dashed box, and the catalytic His25 is depicted as blue area. The semitransparent spheres represent the Hyde score scale, with red spheres indicating positive Hyde scores and green spheres representing negative Hyde scores. The total binding energy (Hyde score) of the WT docking model is displayed below each figure. **c**, A table displaying the estimated affinity ranges for each model. The affinity ranges are represented as horizontal bars.

## Supplementary Figure 5: Comparison of RebD to RebM conversion activity

**a**

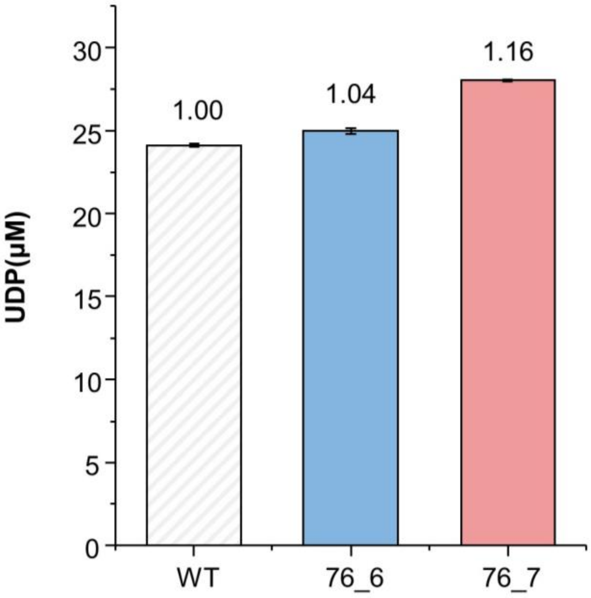


**a**, Glycosyltransferase assay for the conversion of RebD to RebM. 76_6 showed similar conversion activity for RebD to RebM compared to WT. The fold change is shown in each label, relative to the amount of UDP in WT. The error bars represent the standard deviation, calculated based on duplicated measurements.
